# Myeloid Mineralocorticoid Receptor Deficiency Inhibits Aortic Constriction-Induced Cardiac Hypertrophy in Mice

**DOI:** 10.1371/journal.pone.0110950

**Published:** 2014-10-29

**Authors:** Chao Li, Yu Yao Zhang, Ryan A. Frieler, Xiao Jun Zheng, Wu Chang Zhang, Xue Nan Sun, Qing Zhen Yang, Shu Min Ma, Baozhuan Huang, Stefan Berger, Wang Wang, Yong Wu, Ying Yu, Sheng Zhong Duan, Richard M. Mortensen

**Affiliations:** 1 Key Laboratory of Nutrition and Metabolism, Institute for Nutritional Sciences, Shanghai Institutes for Biological Sciences, Chinese Academy of Sciences, Shanghai, China; 2 Department of Molecular and Integrative Physiology, University of Michigan Medical School, Ann Arbor, Michigan, United States of America; 3 Department of Pharmacology, University of Michigan Medical School, Ann Arbor, Michigan, United States of America; 4 Department of Nephrology, Xuhui Central Hospital, Shanghai, China; 5 German Cancer Research Center (DKFZ), Division Molecular Biology of the Cell I, Heidelberg, Germany; 6 Mitochondria and Metabolism Center, Department of Anesthesiology and Pain Medicine, University of Washington, Seattle, Washington, United States of America; 7 Division of Cancer Research and Training, Department of Internal Medicine, Charles R. Drew University of Medicine and Science, Los Angeles, California, United States of America; 8 David Geffen School of Medicine at University of California Los Angeles, Los Angeles, California, United States of America; 9 Department of Internal Medicine, University of Michigan Medical School, Ann Arbor, Michigan, United States of America; Universidad de La Laguna, Spain

## Abstract

Mineralocorticoid receptor (MR) blockade has been shown to suppress cardiac hypertrophy and remodeling in animal models of pressure overload (POL). This study aims to determine whether MR deficiency in myeloid cells modulates aortic constriction-induced cardiovascular injuries. Myeloid MR knockout (MMRKO) mice and littermate control mice were subjected to abdominal aortic constriction (AAC) or sham operation. We found that AAC-induced cardiac hypertrophy and fibrosis were significantly attenuated in MMRKO mice. Expression of genes important in generating reactive oxygen species was decreased in MMRKO mice, while that of manganese superoxide dismutase increased. Furthermore, expression of genes important in cardiac metabolism was increased in MMRKO hearts. Macrophage infiltration in the heart was inhibited and expression of inflammatory genes was decreased in MMRKO mice. In addition, aortic fibrosis and inflammation were attenuated in MMRKO mice. Taken together, our data indicated that MR deficiency in myeloid cells effectively attenuated aortic constriction-induced cardiac hypertrophy and fibrosis, as well as aortic fibrosis and inflammation.

## Introduction

Chronic inflammation is a major feature of many experimental models of heart failure and hypertrophic remodeling [1]. Increased presence of immune cells in myocardium has been identified in many cardiac disease models with maladaptive remodeling, including pressure overload (POL). There is mounting interest in the roles that immune cells play in the pathophysiology of cardiovascular diseases [2]. Modulation of inflammatory signaling has proven to be an effective strategy to regulate cardiac remodeling experimentally [1]. However, the influence that immune cells and inflammation have on ventricular hypertrophy and remodeling during POL remains underappreciated.

Clinical trials have demonstrated beneficial effects of mineralocorticoid receptor (MR) antagonists in treatment of heart failure patients [3]–[5]. Similarly, in rodent models, MR blockade suppressed cardiac hypertrophy and failure induced by POL [6], [7]. However, the underlying mechanisms remain controversial. MR deficiency in cardiomyocytes protects mice from left ventricular dilatation and dysfunction, but not hypertrophy or fibrosis in a POL model [8]. Activation of MR by aldosterone increases the production of reactive oxygen species in blood mononuclear cells and macrophages [9]–[11]. Conditional deletion of MR from myeloid cells induced an alternative macrophage phenotype and demonstrated cardiovascular protection in both angiotensin II (Ang-II)/N(G)-nitro-L-arginine methyl ester (L-NAME) and uninephrectomy/deoxycorticosterone models [12]–[14]. These data indicate that MR in immune cells, particularly myeloid cells, may play a major role in cardiac hypertrophy and fibrosis after POL.

In the present study we used myeloid MR knockout (MMRKO) mice to determine the function of myeloid MR in aortic constriction-induced cardiovascular damages.

## Materials and Methods

### Ethical statement

The studies were carried out in accordance with the NIH Guide for the Care and Use of Laboratory Animals. All animal protocols were approved by the Institutional Animal Care and Use Committee of Institute for Nutritional Sciences, Shanghai Institutes for Biological Sciences, Chinese Academy of Sciences (2012-AN-2) and the University Committee on Use and Care of Animals of the University of Michigan (07798)

### Mice and POL model

Myeloid MR knockout (MMRKO) mice and littermate control (LC) mice were generated as reported before [13]. All mice were in C57BL6/J background and housed in a specific pathogen free (SPF) facility under 12∶12-hour light-dark cycle, fed with standard rodent chow, and given drinking water ad libitum. Male mice (8–10 weeks old, body weight larger than 23 grams) were randomly divided into the following four groups: (1) LC + sham operation (n = 4); (2) LC + abdominal aortic constriction (AAC) (n = 7); (3) MMRKO + sham operation (n = 5); (4) MMRKO + AAC (n = 6). AAC or sham operation was performed as previously described [15]. Briefly, the mice were anesthetized with 2% isoflurane inhalation. Silk sutures (7–0) were used to ligate abdominal aorta against a blunted 27G needle, which was then removed immediately. For sham operations, silk sutures were passed under aortas and then removed without ligation.

### Cardiac hypertrophy estimation

One week after AAC or sham operation, all mice were euthanized using carbon dioxide inhalation and cardiac size was measured as before [16]. Ventricular weight to body weight ratio (VW/BW, mg/g) was used as an indicator of cardiac size. Left ventricles were dissected. Parts of left ventricles close to cardiac base were fixed in formalin and the rest of left ventricles were snap frozen in liquid nitrogen for further analyses.

### Aortic sample collection

Aortas were dissected from aortic arch to the site of ligature. Parts of aortas directly adjacent to the ligatures were fixed in formalin for paraffin sections. The rest of the aortas were cut into halves and the lower halves proximal to the ligatures were snap frozen in liquid nitrogen for RNA extraction.

### Histologic analysis

Formalin-fixed left ventricle or aortic samples were embedded in paraffin and 4 µm sections were stained with hematoxylin and eosin (H&E) or 0.1% picrosirius red [13]. Cardiomyocyte cross-sectional areas were measured as previously described [16]. Fibrotic staining of aortic media and adventitia was quantified as a percentage of stained areas to the total areas examined.

### Analysis of gene expression

Total RNA was isolated using Trizol (Invitrogen), and reverse transcription kits (Takara) were used to synthesize cDNA. qRT-PCR was carried out on an iCycler (Biorad) using SYBR green to detect PCR products. Relative expression of each gene was determined by normalizing to GAPDH for ventricular samples or 18 s for aortic samples.

### Immunofluorescence staining for macrophages

Paraffin sections of left ventricles or aortas were stained for macrophages as previously described [13]. Briefly, after antigen retrieval, the samples were blocked using goat serum and sequentially incubated with primary antibody against Mac2 (eBioscience) and fluorochrome-conjugated secondary antibody (Invitrogen). Fluorescence microscopy images were taken and used for quantitative analysis.

### Statistical analysis

The results were presented as mean ± SE and analyzed using Prism (GraphPad Software). Multiple comparisons were tested with 2-way ANOVA followed by Bonferoni post-tests. Results were considered significantly different if *P* values were ≤0.05.

## Results

### MMRKO protects against aortic constriction-induced cardiac hypertrophy

**Figure 1:**
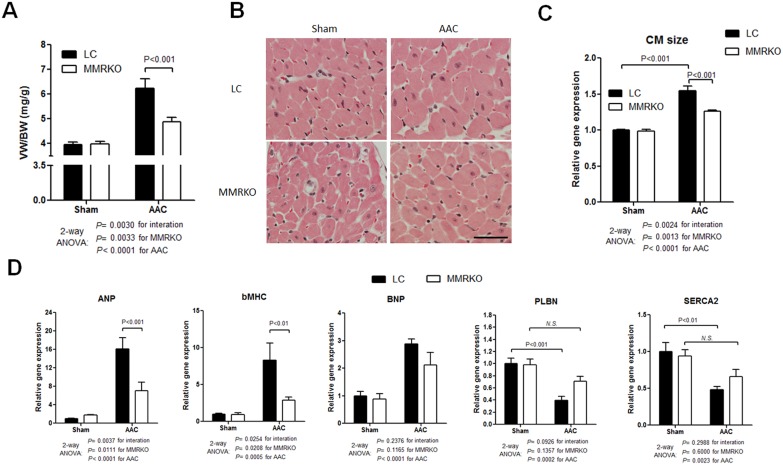
MMRKO protects against aortic constriction-induced cardiac hypertrophy. (A) Ventricular weight to body weight ratio (VW/BW) of littermate control (LC) and myeloid specific MR knockout (MMRKO) mice with or without abdominal aortic constriction (AAC). (B) Representative H&E staining of cross sections of left ventricle samples. Scale bar: 50 µm. (C) Quantification of caridiomyocyte (CM) areas based on the measurements of CM size on H&E stained sections. The results were normalized to those of sham-operated LC group. (D) MMRKO reverses AAC-induced fetal gene program in left ventricles. ANP, Natriuretic peptide A; BNP, Natriuretic peptide B; bMHC, Cardiac myosin heavy chain beta; PLBN, Phospholamban; SERCA 2, Sarcoplasmic reticulum calcium ATPase 2. The expression levels were measured using qRT-PCR. GAPDH was used as an internal control. n = 4–7.

We used MMRKO mice to investigate the role of myeloid MR in the pathological progression of cardiac hypertrophy during POL. Ventricular weight to body weight ratio (VW/BW) demonstrated that MMRKO mice had significantly less cardiac hypertrophy than LC mice after AAC (Figure 1A). H&E staining sections were used to measure the cross-sectional area of cardiomyocytes and the results showed that MMRKO mice had smaller cardiomyocytes than LC after AAC (Figure 1B, C). Pathological cardiac hypertrophy is usually accompanied by a fetal gene program, including increases in ANP, BNP, and bMHC and decrease in PLBN and SERCA2 [17]. qRT-PCR analysis revealed that expression of both ANP and bMHC was significantly suppressed in the ventricular samples from MMRKO compared with LC mice (Figure 1D). Expression of both PLBN and SERCA2 was significantly down-regulated by AAC in the ventricular samples from LC mice but not in those from MMRKO mice, further suggesting that MMRKO may play a protective role.

### MMRKO protects against POL-induced cardiac fibrosis

**Figure 2:**
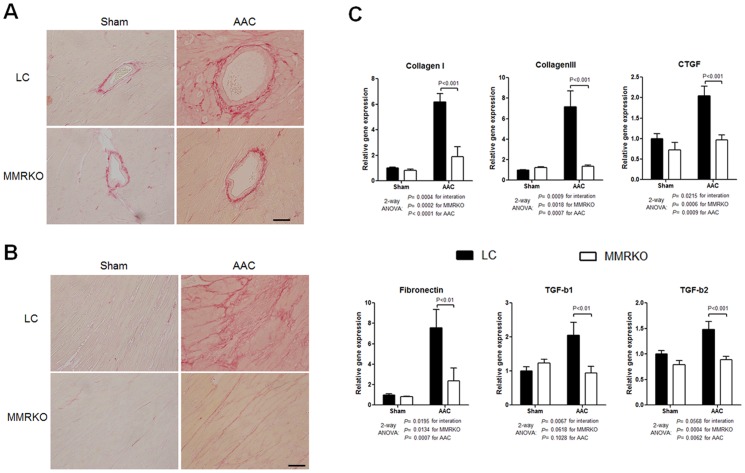
MMRKO improves aortic constriction-induced cardiac fibrosis. (A) and (B) Representative picrosirius red staining of heart sections showing perivascular and interstitial fibrosis respectively. Scale bar: 50 µm. (C) MMRKO attenuates up-regulation of fibrotic genes induced by AAC in left ventricles. CTGF, Connective tissue growth factor precursor; TGFb1, Transforming growth factor-beta 1; TGFb2, Transforming growth factor-beta 2. The expression levels were measured using qRT-PCR. GAPDH was used as an internal control. n = 4–7.

Cardiac fibrosis is an important process in cardiac remodeling after injury. The results of picrosirius red staining showed that MMRKO mice had dramatically less perivascular and interstitial fibrosis (Figure 2A, B). Accordingly, MMRKO significantly inhibited the expression of fibrosis-related genes such as Collagen I, Collagen III, CTGF, fibronectin 1, TGF-b1, and TGF-b2 (Figure 2C).

### Effects of MMRKO on gene expression involved in oxidative stress and cardiac metabolism

**Figure 3:**
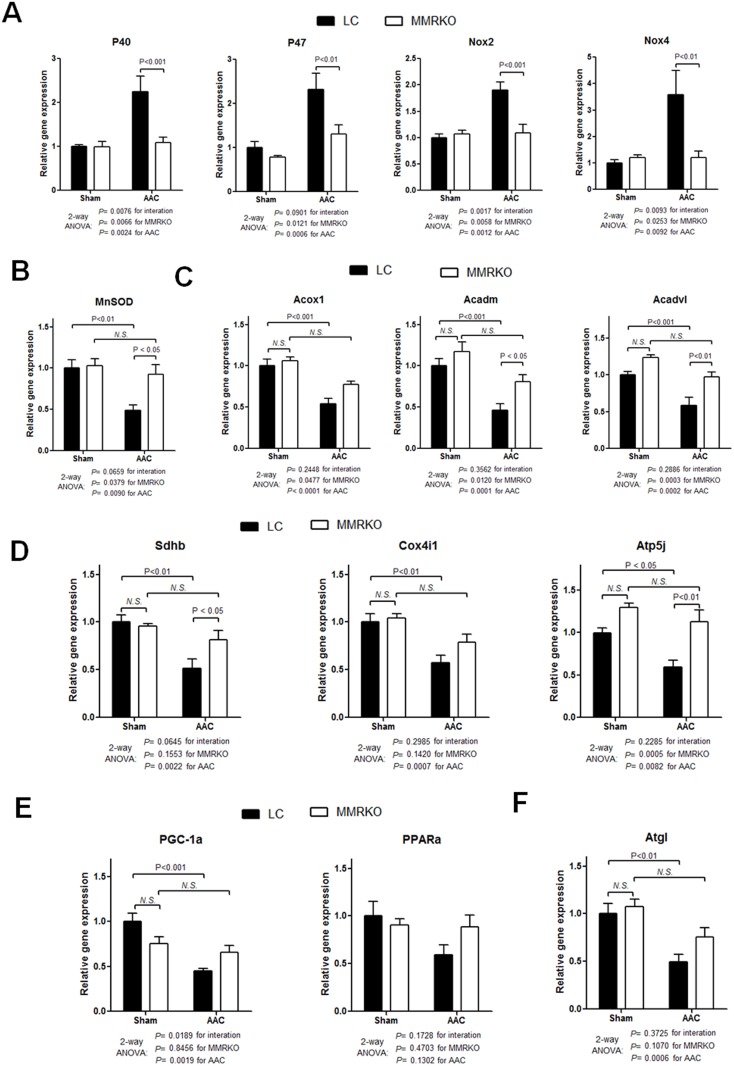
Effects of MMRKO on expression of genes related to oxidative stress and cardiac metabolism. (A) MMRKO decreases expression of genes important in generating reactive oxygen species. P40, p40phox; p47, p47phox; NOX2, NADPH oxidase 2; NOX4, NADPH oxidase 4. (B) MMRKO increases expression of manganese superoxide dismutase (MnSOD). (C) (D) (E) and (F) MMRKO upregulates expression of genes involved in cardiac metabolism. PPARa, peroxisome proliferator-activated receptor alpha; PGC-1a, peroxisome proliferator-activated receptor gamma coactivator 1-alpha; Acox1, acyl-Coenzyme A oxidase 1; Acadm, acyl-Coenzyme A dehydrogenase, medium chain; Acadvl, acyl-Coenzyme A dehydrogenase, very long chain; Sdhb, succinate dehydrogenase complex, subunit B, iron sulfur (Ip); Cox4i1, cytochrome c oxidase subunit IV isoform 1; Atp5j, ATP synthase, H+ transporting, mitochondrial F0 complex, subunit F; ATGL, adipose triglyceride lipase; OXPHOX, oxidative phosphorylation. The expression levels were measured using qRT-PCR. GAPDH was used as an internal control. n = 4–7.

Elevated reactive oxygen species (ROS) are associated with cardiac hypertrophy [18], [19]. NADPH oxidases (Nox) are important sources of ROS in the heart [18], [19]. We found that the expression of the catalytic subunits, Nox2 and Nox4, as well as the regulatory subunits, p40^phox^ and p47^phox^, was elevated in left ventricles after AAC. However, the induction of the expression of these genes was attenuated in MMRKO mice (Figure 3A). Conversely, gene expression of manganese superoxide dismutase (MnSOD), the mitochondrial superoxide dismutase that plays a key role in scavenging mitochondrial ROS, was decreased in left ventricles of LC mice but not MMRKO mice after AAC. As a result, the expression of MnSOD in left ventricles of MMRKO mice was significantly higher than that of LC mice (Figure 3B).

Alterations in myocardial metabolism, such as increased glucose metabolism and decreased fatty acid oxidation, are characteristic of cardiac hypertrophy. Hypertrophied hearts typically demonstrate decreased gene expression for β-oxidation and oxidative phosphorylation [20]. Consistent with this notion, we found that expression of genes related to β-oxidation (Acox1, Acadm and Acadvl) and oxidative phosphorylation (Sdhb, Cox4il, Atp5j) was decreased in LC mice after AAC. However, the expression of these genes was much higher in the left ventricles of MMRKO mice after AAC, indicating less suppressed cardiac metabolism (Figure 3C and 3D). There was a trend that gene expression of PPARa and PGC-1a, two central transcriptional regulators in fatty acid metabolism, was improved in MMRKO mice after AAC (Figure 3E). Adipose triglyceride lipase (Atgl) is necessary to maintain normal metabolism in the heart [21]. We found that the expression of Atgl was significantly deceased in left ventricles of LC mice but not MMRKO mice after AAC (Figure 3F)

### MMRKO reduces aortic constriction-induced cardiac inflammation

**Figure 4:**
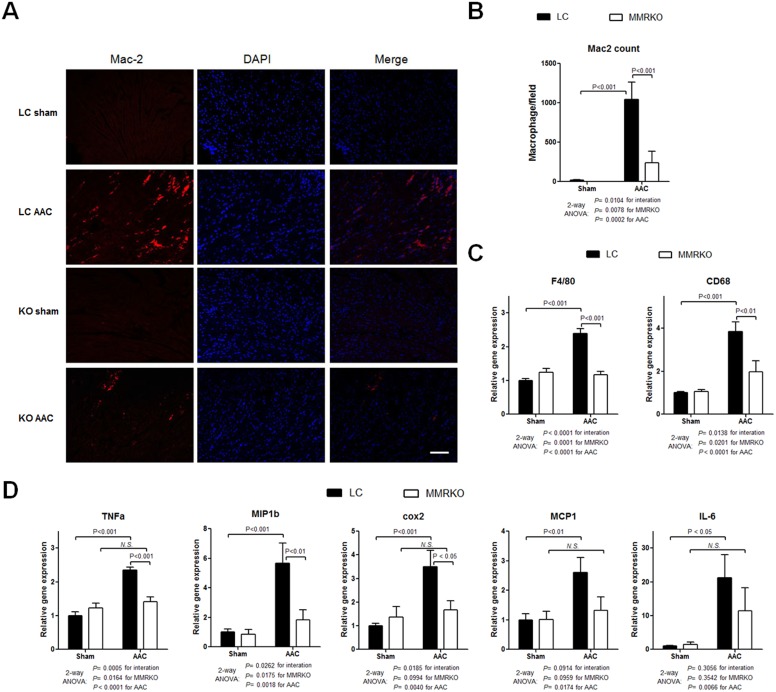
MMRKO reduces aortic constriction-induced cardiac inflammation. (A) Representative immunofluorescence assay of cross sections of heart samples. Mac2 antibody was used to detect macrophages. Scale bar: 100 µm. (B) Quantification of macrophages stained by Mac2. (C) MMRKO decreases expression of macrophage marker genes in left ventricles. F4/80 (Emr1), EGF-like module containing, mucin-like, hormone receptor-like sequence 1. (D) MMRKO decreases expression of inflammatory genes in left ventricles. TNFa, tumor necrosis factor alpha; MIP-1b (Ccl4), chemokine (C-C motif) ligand 4; Cox2 (Ptgs2), prostaglandin-endoperoxide 2; MCP-1 (Ccl2), chemokine (C-C motif) ligand 2; IL-6, interleukin 6. The expression levels were measured using qRT-PCR. GAPDH was used as an internal control. n = 4–7.

Inflammation is closely involved in ventricular remodeling in response to POL [22], [23]. Macrophage accumulation and cytokine secretion are two common features of chronic inflammation. We used Mac-2 antibody to detect macrophages in left ventricles. As shown in Figure 4A and B, MMRKO significantly decreased macrophage accumulation induced by AAC. Accordingly, the expression of macrophage marker genes, F4/80 and CD68, was also decreased in left ventricles of MMRKO mice compared with LC mice (Figure 4C). AAC induced expression of inflammatory genes, including TNFa, MIP1b, Cox2, MCP1, and IL-6, in the left ventricles of LC mice. In comparison, the expression of TNFa, MIP1b, and Cox2 was significantly attenuated in the left ventricles of MMRKO mice (Figure 4D). These results suggest that MMRKO may protect against AAC-induced cardiac inflammation.

### MMRKO protects against aortic constriction-induced aortic fibrosis

**Figure 5:**
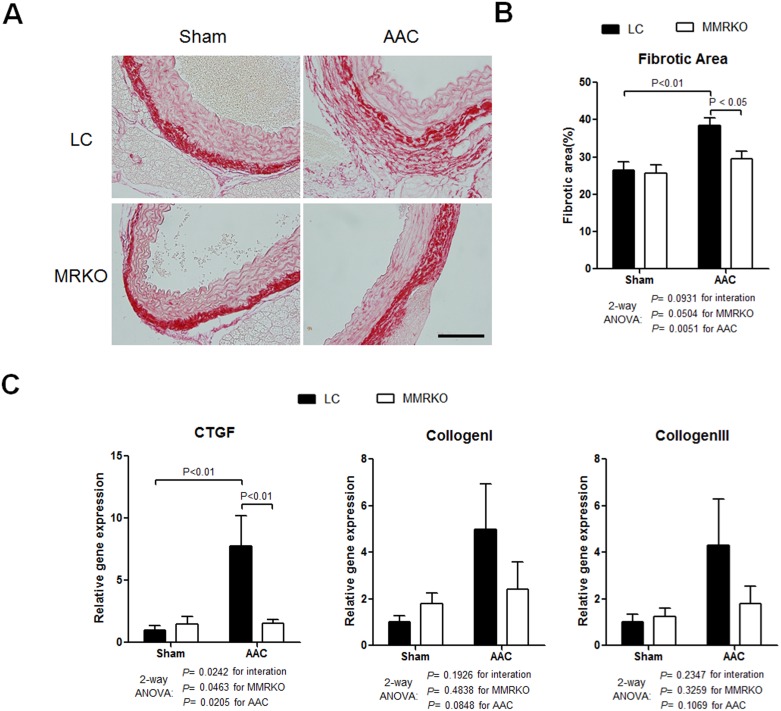
MMRKO improves aortic constriction-induced aortic fibrosis. (A) Representative picrosirius red staining of aortic sections showing vascular fibrosis. Scale bar: 100 µm. (B) Quantification of fibrotic area stained by picrosirius red. (C) MMRKO attenuates up-regulation of fibrotic genes induced by AAC in aortas. CTGF, Connective tissue growth factor precursor. The expression levels were measured using qRT-PCR. 18 s was used as an internal control. n = 3–5.

AAC also induced significant fibrosis in aortas. MMRKO mice had less fibrotic areas in aortas compared to LC after ACC (Figure 5A and 5B). Accordingly, expression of fibrotic gene, CTGF, was significantly inhibited in MMRKO aortas. Expression of Collagen I and Collagen III followed similar trend in MMRKO aortas (Figure 5C).

### Aortic inflammation is mildly attenuated in MMRKO mice

**Figure 6:**
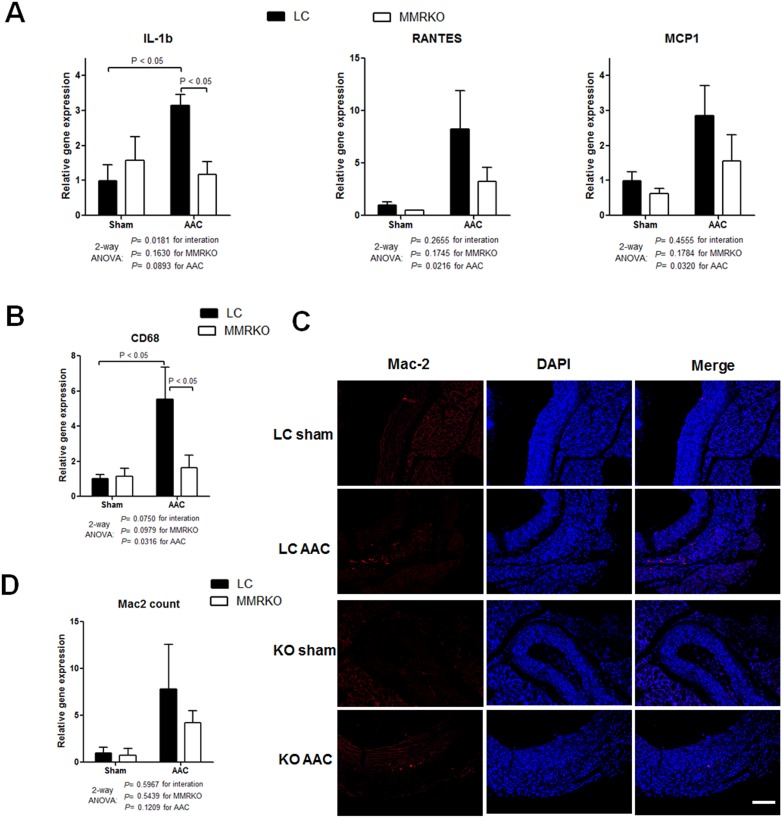
MMRKO mildly improves aortic constriction-induced aortic inflammation. (A) Effects of MMRKO on expression of inflammatory genes. (B) MMRKO decreases expression of macrophage marker gene CD68 in aortas. The expression levels were measured using qRT-PCR. 18 s was used as an internal control. n = 3–5. (C) Representative immunofluorescence assay of aortas. Scale bar: 100 µm. (D) Quantification of macrophages stained by Mac2.

To investigate whether MMRKO has an effect on aortic inflammation after AAC, we measured expression of inflammatory genes and macrophage accumulation in aortas. Expression of IL-1b was significantly inhibited in aortas of MMRKO mice, although that of other inflammatory genes such as RANTES and MCP-1 was not significantly changed (Figure 6A). Expression of CD68, a macrophage maker gene, was significantly suppressed in aortas of MMRKO mice (Figure 6B). However, macrophage accumulation was not significantly different between aortas of MMRKO and LC mice (Figure 6C and 6D).

## Discussion

Research on MR in recent years has demonstrated that this classic nuclear receptor plays important roles in cardiovascular system beyond its traditional role in regulating electrolyte homeostasis and blood pressure [24], [25]. Particularly, studies have started to identify the potential targeting cells of MR antagonists and the detailed mechanisms behind their effects. Data from cell type-specific knockout mouse models revealed that deletion of MR in myeloid cells, cardiomyocytes, endothelial cells, or vascular smooth muscle cells in general protects mice from cardiovascular disease [12], [13], [26]–[30]. Mechanistically, oxidative stress and inflammation are recurring themes of MR actions in cardiovascular system [25], [31].

Several previous reports have demonstrated the importance of MR in POL-induced cardiovascular damages. Kuster et al. demonstrated that eplerenone decreased cardiac fibrosis and improved cardiac function in a model of aortic constriction [6]. These improvements were associated with decreased myocardial oxidative stress and inflammation but not with blood pressure alterations. Subsequently, Nagata et al. showed that eplerenone attenuated cardiac hypertrophy and heart failure in hypertensive rats without changing blood pressure [7]. Similarly, these effects were associated with decreased oxidative stress and inflammation. Further, cell type-specific knockout mouse models were used to delineate the importance of myeloid MR in cardiovascular system. Our previous work demonstrated that MMRKO protected against cardiac and vascular hypertrophy, inflammation, and fibrosis induced by L-NAME/Ang-II^13^. Similar effects of MMRKO on cardiac fibrosis and inflammation were reported by other researchers using mouse models that combined uninephrectomy/salt with deoxycorticosterone or L-NAME [12], [14]. These studies together established the protective roles of myeloid MR deficiency in POL-induced cardiovascular damages. In the current study, we further demonstrated that MR deficiency in myeloid cells attenuated cardiac hypertrophy and fibrosis, as well as aortic fibrosis in a mouse model of aortic constriction-induced cardiovascular damages.

This study illustrated the impact of MMRKO on expression of genes related to oxidative stress. On one hand, MMRKO prevented the AAC-induced expression of Nox2, Nox4, and their regulatory subunits, p40^phox^ and p47^phox^. On the other hand, MMRKO increased MnSOD expression. These results suggest that during POL, there may be a decreased ROS production or preserved ROS scavenging in the left ventricles of MMRKO mice. Cardiomyocyte-specific MRKO prevented the up-regulation of Nox2, Nox4 and ROS induced by myocardial infarction, suggesting that cardiomyocyte MR is important in regulating oxidative stress [28]. Myeloid cells including neutrophils and macrophages also express Nox, which are important sources of ROS [32]. It remains to be further clarified in which cell type MR plays more important roles in regulating the expression of genes related to ROS production or scavenging. Furthermore, if myeloid cells are the major player, it would be interesting to further investigate whether the quantity of infiltrated macrophages or the quality of each individual cell is the major determinant.

Pathological cardiac hypertrophy is usually accompanied by abnormal cardiac metabolism that eventually contributes to the maladaptation and heart failure. Particularly, severely suppressed fatty acid oxidation leads to a decreased supply of adenosine triphosphate (ATP) [33]. Our data suggest that MMRKO improves the expression of genes related to fatty acid metabolism in the heart under AAC. The improvement of the expression of these genes is correlated with attenuated cardiac hypertrophy and remodeling.

Inflammation is an important contributor to cardiovascular remodeling. Our previous work^13^ showed that macrophages lacking MR exhibited a profile of alternatively activation. In the current study, we found that MMRKO decreased inflammation both in hearts and aortas during AAC. Such anti-inflammatory effects of macrophages lacking MR are correlated with attenuated cardiac hypertrophy and fibrosis, as well as attenuated aortic fibrosis.

In the process of pathological cardiac hypertrophy and heart failure, there exist intertwined relationships among oxidative stress, inflammation, and deterioration of cardiac metabolism. First, oxidative stress and inflammation form a ‘vicious’ perpetuating cycle [34]. ROS stimulates the release of inflammatory cytokines by activating downstream pathways involving activator protein 1, nuclear factor kappa B, and mitogen-activated protein kinases. Conversely, pro-inflammatory cytokines such as TNFα suppress antioxidants while stimulating pro-oxidants. By contrast, anti-inflammatory cytokines such as IL-10 stimulate antioxidants while suppress pro-oxidants. Second, oxidative stress and cardiac metabolism affect each other [35]. ROS causes mitochondria dysfunction in cardiomyocytes and then affects the fatty acid metabolism of the heart. Altered cardiac metabolism, in turn, increases the production of ROS. The ineffectiveness of anti-oxidants or anti-inflammatory agents in treating heart failure reflects the complexity of the pathophysiology of cardiac hypertrophy and heart failure [34]. Our results demonstrated that MR deficiency in myeloid cells, particularly macrophages, resulted in inhibition of cardiovascular damages induced by AAC. Further, these beneficial effects of MR deficiency were associated with improved profile of gene expression related to cardiac metabolism, oxidative stress, and inflammation. These data suggest that blockade of MR in myeloid cells may present as a potentially fruitful intervention for cardiovascular damages in the setting of POL. However, more studies are needed to test potential causative relationships between cardiovascular protection and improvement on cardiac metabolism, oxidative stress, and inflammation. Future work is required to differentiate direct and indirect functions of myeloid MR during cardiovascular damages. It is also important to explore the molecular mechanisms how MR affects the functions of macrophages.

## Supporting Information

Checklist S1 **ARRIVE Checklist.**
(DOC)
