# Supplementary material for: Myeloid Mineralocorticoid Receptor Deficiency Inhibits Aortic Constriction-Induced Cardiac Hypertrophy in Mice
Source: PLoS One. 2014 Oct 29;9(10):e110950. doi: 10.1371/journal.pone.0110950 (PMC4212990; doi:10.1371/journal.pone.0110950)
Supplement: Checklist S1 — ARRIVE Checklist. (DOC) [file pone.0110950.s001.doc]

**The ARRIVE Checklist**

**TITLE**

**1 Provide as accurate and concise a description of the content of the article as possible.**

Myeloid mineralocorticoid receptor deficiency inhibits aortic constriction-induced cardiac hypertrophy in mice

**ABSTRACT**

**2 Provide an accurate summary of the background, research objectives (including details of the species or strain of animal used), key methods, principal findings, and conclusions of the study.**

Mineralocorticoid receptor (MR) blockade has been shown to suppress cardiac hypertrophy and remodeling in animal models of pressure overload (POL). This study aims to determine whether MR deficiency in myeloid cells modulates aortic constriction-induced cardiovascular injuries. Myeloid MR knockout (MMRKO) mice and littermate control mice were subjected to abdominal aortic constriction (AAC) or sham operation. We found that AAC-induced cardiac hypertrophy and fibrosis were significantly attenuated in MMRKO mice. Expression of genes important in generating reactive oxygen species was decreased in MMRKO mice, while that of manganese superoxide dismutase increased. Furthermore, expression of genes important in cardiac metabolism was increased in MMRKO hearts. Macrophage infiltration in the heart was inhibited and expression of inflammatory genes was decreased in MMRKO mice. In addition, aortic fibrosis and inflammation were attenuated in MMRKO mice. Taken together, our data indicated that MR deficiency in myeloid cells effectively attenuated aortic constriction-induced cardiac hypertrophy and fibrosis, as well as aortic fibrosis and inflammation.

**INTRODUCTION**

**Background**

**3 a. Include sufficient scientific background (including relevant references to previous work) to understand the motivation and context for the study, and explain the experimental approach and rationale.**

**b. Explain how and why the animal species and model being used can address the scientific objectives and, where appropriate, the study’s relevance to human biology.**

Chronic inflammation is a major feature of many experimental models of heart failure and hypertrophic remodeling. Increased presence of immune cells in myocardium has been identified in many cardiac disease models with maladaptive remodeling, including pressure overload (POL). There is mounting interest in the roles that immune cells play in the pathophysiology of cardiovascular diseases. Modulation of inflammatory signaling has proven to be an effective strategy to regulate cardiac remodeling experimentally. However, the influence that immune cells and inflammation have on ventricular hypertrophy and remodeling during POL remains underappreciated.

Clinical trials have demonstrated beneficial effects of mineralocorticoid receptor (MR) antagonists in treatment of heart failure patients. Similarly, in rodent models, MR blockade suppressed cardiac hypertrophy and failure induced by POL. However, the underlying mechanisms remain controversial. MR deficiency in cardiomyocytes protects mice from left ventricular dilatation and dysfunction, but not hypertrophy or fibrosis in a POL model. Activation of MR by aldosterone increases the production of reactive oxygen species in blood mononuclear cells and macrophages. Conditional deletion of MR from myeloid cells induced an alternative macrophage phenotype and demonstrated cardiovascular protection in both angiotensin II (Ang-II)/N(G)-nitro-L-arginine methyl ester (L-NAME) and uninephrectomy/deoxycorticosterone models. These data indicate that MR in immune cells, particularly myeloid cells, may play a major role in cardiac hypertrophy and fibrosis after POL.

In the present study we used myeloid MR knockout (MMRKO) mice to determine the function of myeloid MR in aortic constriction-induced cardiovascular damages.

**Objectives**

**4 Clearly describe the primary and any secondary objectives of the study, or specific hypotheses being tested.**

- To determine whether MR deficiency in myeloid cells modulates aortic constriction-induced cardiovascular damage.

**METHODS**

**Ethical statement**

**5 Indicate the nature of the ethical review permissions, relevant licenses (e.g. Animal [Scientific Procedures] Act 1986), and national or institutional guidelines for the care and use of animals, that cover the research.**

The studies were carried out in accordance with the NIH Guide for the Care and Use of Laboratory Animals. All animal protocols were approved by the Institutional Animal Care and Use Committee of Institute for Nutritional Sciences, Shanghai Institutes for Biological Sciences, Chinese Academy of Sciences (2012-AN-2) and the University Committee on Use and Care of Animals of the University of Michigan (07798).

**Study design**

**6 For each experiment, give brief details of the study design, including:**

**a. The number of experimental and control groups.**

**b. Any steps taken to minimise the effects of subjective bias when allocating animals to treatment (e.g., randomisation procedure) and when assessing results (e.g., if done, describe who was blinded and when).**

**c. The experimental unit (e.g. a single animal, group, or cage of animals).**

**A time-line diagram or flow chart can be useful to illustrate how complex study designs were carried out.**

1. Our experiment includes four groups: (1) LC + sham operation (n=4); (2) LC + abdominal aortic constriction (AAC) (n=7); (3) MMRKO + sham operation (n=5); (4) MMRKO + AAC (n=6).
2. The mice were randomly divided into groups.
3. The experimental unit is group.

**Experimental procedures**

**7 For each experiment and each experimental group, including controls, provide precise details of all procedures carried out. For example:**

**a. How (e.g., drug formulation and dose, site and route of administration, anaesthesia and analgesia used [including monitoring], surgical procedure, method of euthanasia). Provide details of any specialist equipment used, including supplier(s).**

**b. When (e.g., time of day).**

**c. Where (e.g., home cage, laboratory, water maze).**

**d. Why (e.g., rationale for choice of specific anaesthetic, route of administration, drug dose used).**

**Animal model:** Male mice (8-10 weeks old, body weight larger than 23 grams) were randomly divided into the following four groups: (1) LC + sham operation (n=4); (2) LC + abdominal aortic constriction (AAC) (n=7); (3) MMRKO + sham operation (n=5); (4) MMRKO + AAC (n=6). AAC or sham operation was performed as previously described15. Briefly, the mice were anesthetized with 2% isoflurane inhalation. Silk sutures (7–0) were used to ligate abdominal aorta against a blunted 27G needle, which was then removed immediately. For sham operations, silk sutures were passed under aortas and then removed without ligation.

**Cardiac hypertrophy estimation:** One week after AAC or sham operation, all mice were euthanized using carbon dioxide inhalation and cardiac size was measured as before. Ventricular weight to body weight ratio (VW/BW, mg/g) was used as an indicator of cardiac size. Left ventricles were dissected. Parts of left ventricles close to cardiac base were fixed in formalin and the rest of left ventricles were snap frozen in liquid nitrogen for further analyses.

**Aortic sample collection:** Aortas were dissected from aortic arch to the site of ligature. Parts of aortas directly adjacent to the ligatures were fixed in formalin for paraffin sections. The rest of the aortas were cut into halves and the lower halves proximal to the ligatures were snap frozen in liquid nitrogen for RNA extraction.

**Histologic analysis.**: Formalin-fixed left ventricle or aortic samples were embedded in paraffin and 4μm sections were stained with hematoxylin and eosin (H&E) or 0.1% picrosirius red. Cardiomyocyte cross-sectional areas were measured. Fibrotic staining of aortic media and adventitia was quantified as a percentage of stained areas to the total areas examined.

**Analysis of gene expression:** Total RNA was isolated using Trizol (Invitrogen), and reverse transcription kits (Takara) were used to synthesize cDNA. qRT-PCR was carried out on an iCycler (Biorad) using SYBR green to detect PCR products. Relative expression of each gene was determined by normalizing to GAPDH for ventricular samples or 18s for aortic samples.

Immunofluorescence staining for macrophages. Paraffin sections of left ventricles or aortas were stained for macrophages. Briefly, after antigen retrieval, the samples were blocked using goat serum and sequentially incubated with primary antibody against Mac2 (eBioscience) and fluorochrome-conjugated secondary antibody (Invitrogen). Fluorescence microscopy images were taken and used for quantitative analysis.

**Experimental animals**

**8 a. Provide details of the animals used, including species, strain, sex, developmental stage (e.g., mean or median age plus age range), and weight (e.g., mean or median weight plus weight range).**

**b. Provide further relevant information such as the source of animals, international strain nomenclature, genetic modification status (e.g. knock-out or transgenic), genotype, health/immune status, drug- or test naıve, previous procedures, etc.**

Myeloid MR knockout (MMRKO) mice and littermate control (LC) mice were generated as reported before. All mice were in C57BL6/J background and housed in a specific pathogen free (SPF) facility under 12:12-hour light-dark cycle, fed with standard rodent chow, and given drinking water ad libitum. Male mice (8-10 weeks old, body weight larger than 23 grams) were randomly divided into four groups.

**Housing and husbandry**

**9 Provide details of:**

**a. Housing (e.g., type of facility, e.g., specific pathogen free (SPF); type of cage or housing; bedding material; number of cage companions; tank shape and material etc. for fish).**

**b. Husbandry conditions (e.g., breeding programme, light/dark cycle, temperature, quality of water etc. for fish, type of food, access to food and water, environmental enrichment).**

**c. Welfare-related assessments and interventions that were carried out before, during, or after the experiment.**

All mice were housed in a specific pathogen free (SPF) facility under 12:12-hour light-dark cycle, fed with standard rodent chow, and given drinking water ad libitum.

**Sample size**

**10 a. Specify the total number of animals used in each experiment and the number of animals in each experimental group.**

**b. Explain how the number of animals was decided. Provide details of any sample size calculation used.**

**c. Indicate the number of independent replications of each experiment, if relevant.**

Male mice (8-10 weeks old, body weight larger than 23 grams) were randomly divided into the following four groups: (1) LC + sham operation (n=4); (2) LC + abdominal aortic constriction (AAC) (n=7); (3) MMRKO + sham operation (n=5); (4) MMRKO + AAC (n=6).

**Allocating animals to experimental groups**

**11 a. Give full details of how animals were allocated to experimental groups, including randomisation or matching if done.**

**b. Describe the order in which the animals in the different experimental groups were treated and assessed.**

Male mice (8-10 weeks old, body weight larger than 23 grams) were randomly divided into four groups.

**Experimental outcomes**

**12 Clearly define the primary and secondary experimental outcomes assessed (e.g., cell death, molecular markers, behavioural changes).**

Cardiac hypertrophy and fibrosis, gene expression, macrophage staining, as well as vascular fibrosis, gene expression and macrophage staining are the experimental outcomes.

**Statistical methods**

**13 a. Provide details of the statistical methods used for each analysis.**

**b. Specify the unit of analysis for each dataset (e.g. single animal, group of animals, single neuron).**

**c. Describe any methods used to assess whether the data met the assumptions of the statistical approach.**

The results were presented as mean ± SE and analyzed using Prism (GraphPad Software). Multiple comparisons were tested with 2-way ANOVA followed by Bonferoni post-tests. Results were considered significantly different if P values were ≤ 0.05.

**RESULTS**

**Baseline data
14 For each experimental group, report relevant characteristics and health status of animals (e.g., weight, microbiological status, and drug- or test-naıve) before treatment or testing (this information can often be tabulated).**

All animals analyzed were in good health.

**Numbers analysed**

**15 a. Report the number of animals in each group included in each analysis. Report absolute numbers (e.g. 10/20, not 50%).**

**b. If any animals or data were not included in the analysis, explain why.**

Please refer to #10.

**Outcomes and estimation**

**16 Report the results for each analysis carried out, with a measure of precision (e.g., standard error or confidence interval).**

Please refer to #13.

**Adverse events**

**17 a. Give details of all important adverse events in each experimental group.**

**b. Describe any modifications to the experimental protocols made to reduce adverse events.**

There were no adverse events.

**DISCUSSION**

**Interpretation/scientific implications**

**18 a. Interpret the results, taking into account the study objectives and hypotheses, current theory, and other relevant studies in the literature.**

**b. Comment on the study limitations including any potential sources of bias, any limitations of the animal model, and the imprecision associated with the results.**

**c. Describe any implications of your experimental methods or findings for the replacement, refinement, or reduction (the 3Rs) of the use of animals in research.**

Research on MR in recent years has demonstrated that this classic nuclear receptor plays important roles in cardiovascular system beyond its traditional role in regulating electrolyte homeostasis and blood pressure. Particularly, studies have started to identify the potential targeting cells of MR antagonists and the detailed mechanisms behind their effects. Data from cell type-specific knockout mouse models revealed that deletion of MR in myeloid cells, cardiomyocytes, endothelial cells, or vascular smooth muscle cells in general protects mice from cardiovascular disease. Mechanistically, oxidative stress and inflammation are recurring themes of MR actions in cardiovascular system.

Several previous reports have demonstrated the importance of MR in POL-induced cardiovascular damages. Kuster et al. demonstrated that eplerenone decreased cardiac fibrosis and improved cardiac function in a model of aortic constriction. These improvements were associated with decreased myocardial oxidative stress and inflammation but not with blood pressure alterations. Subsequently, Nagata et al. showed that eplerenone attenuated cardiac hypertrophy and heart failure in hypertensive rats without changing blood pressure. Similarly, these effects were associated with decreased oxidative stress and inflammation. Further, cell type-specific knockout mouse models were used to delineate the importance of myeloid MR in cardiovascular system. Our previous work demonstrated that MMRKO protected against cardiac and vascular hypertrophy, inflammation, and fibrosis induced by L-NAME/Ang-II. Similar effects of MMRKO on cardiac fibrosis and inflammation were reported by other researchers using mouse models that combined uninephrectomy/salt with deoxycorticosterone or L-NAME. These studies together established the protective roles of myeloid MR deficiency in POL-induced cardiovascular damages. In the current study, we further demonstrated that MR deficiency in myeloid cells attenuated cardiac hypertrophy and fibrosis, as well as aortic fibrosis in a mouse model of aortic constriction-induced cardiovascular damages.

This study illustrated the impact of MMRKO on expression of genes related to oxidative stress. On one hand, MMRKO prevented the AAC-induced expression of Nox2, Nox4, and their regulatory subunits, p40phox and p47phox. On the other hand, MMRKO increased MnSOD expression. These results suggest that during POL, there may be a decreased ROS production or preserved ROS scavenging in the left ventricles of MMRKO mice. Cardiomyocyte-specific MRKO prevented the up-regulation of Nox2, Nox4 and ROS induced by myocardial infarction, suggesting that cardiomyocyte MR is important in regulating oxidative stress. Myeloid cells including neutrophils and macrophages also express Nox, which are important sources of ROS. It remains to be further clarified in which cell type MR plays more important roles in regulating the expression of genes related to ROS production or scavenging. Furthermore, if myeloid cells are the major player, it would be interesting to further investigate whether the quantity of infiltrated macrophages or the quality of each individual cell is the major determinant.

Pathological cardiac hypertrophy is usually accompanied by abnormal cardiac metabolism that eventually contributes to the maladaptation and heart failure. Particularly, severely suppressed fatty acid oxidation leads to a decreased supply of adenosine triphosphate (ATP). Our data suggest that MMRKO improves the expression of genes related to fatty acid metabolism in the heart under AAC. The improvement of the expression of these genes is correlated with attenuated cardiac hypertrophy and remodeling.

Inflammation is an important contributor to cardiovascular remodeling. Our previous work showed that macrophages lacking MR exhibited a profile of alternatively activation. In the current study, we found that MMRKO decreased inflammation both in hearts and aortas during AAC. Such anti-inflammatory effects of macrophages lacking MR are correlated with attenuated cardiac hypertrophy and fibrosis, as well as attenuated aortic fibrosis.

In the process of pathological cardiac hypertrophy and heart failure, there exist intertwined relationships among oxidative stress, inflammation, and deterioration of cardiac metabolism. First, oxidative stress and inflammation form a ‘vicious’ perpetuating cycle. ROS stimulates the release of inflammatory cytokines by activating downstream pathways involving activator protein 1, nuclear factor kappa B, and mitogen-activated protein kinases. Conversely, pro-inflammatory cytokines such as TNFα suppress antioxidants while stimulating pro-oxidants. By contrast, anti-inflammatory cytokines such as IL-10 stimulate antioxidants while suppress pro-oxidants. Second, oxidative stress and cardiac metabolism affect each other. ROS causes mitochondria dysfunction in cardiomyocytes and then affects the fatty acid metabolism of the heart. Altered cardiac metabolism, in turn, increases the production of ROS. The ineffectiveness of anti-oxidants or anti-inflammatory agents in treating heart failure reflects the complexity of the pathophysiology of cardiac hypertrophy and heart failure. Our results demonstrated that MR deficiency in myeloid cells, particularly macrophages, resulted in inhibition of cardiovascular damages induced by AAC. Further, these beneficial effects of MR deficiency were associated with improved profile of gene expression related to cardiac metabolism, oxidative stress, and inflammation. These data suggest that blockade of MR in myeloid cells may present as a potentially fruitful intervention for cardiovascular damages in the setting of POL. However, more studies are needed to test potential causative relationships between cardiovascular protection and improvement on cardiac metabolism, oxidative stress, and inflammation. Future work is required to differentiate direct and indirect functions of myeloid MR during cardiovascular damages. It is also important to explore the molecular mechanisms how MR affects the functions of macrophages.

**Generalisability/translation**

**19 Comment on whether, and how, the findings of this study are likely to translate to other species or systems, including any relevance to human biology.**

Our results demonstrated that MR deficiency in myeloid cells, particularly macrophages, resulted in inhibition of cardiovascular damages induced by AAC. Further, these beneficial effects of MR deficiency were associated with improved profile of gene expression related to cardiac metabolism, oxidative stress, and inflammation. These data suggest that blockade of MR in myeloid cells may present as a potentially fruitful intervention for cardiovascular damages in the setting of POL.

**Funding**

**20 List all funding sources (including grant number) and the role of the funder(s) in the study.**

This work was supported by grants from the One Hundred Talents Program of the Chinese Academy of Sciences (2012OHTP06), the Ministry of Science and Technology of China (973 Program 2012CB524900), the National Natural Science Foundation of China (31371153, 91339110, 31171133), the Knowledge Innovation Program of the Chinese Academy of Sciences (KSCX2-EW-R-08), and Xuhui Central Hospital, Shanghai, China (CRC2011003) (to S.Z. Duan), as well as grants from the National Heart, Lung, and Blood Institute, NIH (R01HL083201) and American Diabetes Association (1-08-RA-137) (to R.M. Mortensen). The funders had no role in study design, data collection and analysis, decision to publish, or preparation of the manuscript.
